# Antiviral, Cytoprotective, and Anti-Inflammatory Effect of *Ampelozizyphus amazonicus* Ducke Ethanolic Wood Extract on Chikungunya Virus Infection

**DOI:** 10.3390/v15112232

**Published:** 2023-11-09

**Authors:** Daniele C. P. Rocha, Tháyna Sisnande, Daniel Gavino-Leopoldino, Iris Paula Guimarães-Andrade, Fernanda F. Cruz, Iranaia Assunção-Miranda, Simony C. Mendonça, Gilda Guimarães Leitão, Rosineide Costa Simas, Ronaldo Mohana-Borges, Suzana Guimarães Leitão, Diego Allonso

**Affiliations:** 1 Departamento de Biotecnologia Farmacêutica, Faculdade de Farmácia, Universidade Federal do Rio de Janeiro, Rio de Janeiro 21941-902, RJ, Brazil; danielecp@biof.ufrj.br; 2 Laboratório de Biotecnologia e Bioengenharia Estrutural, Instituto de Biofísica Carlos Chagas Filho, Universidade Federal do Rio de Janeiro, Rio de Janeiro 21941-902, RJ, Brazil; thayna.sisnande@biof.ufrj.br (T.S.); mohana@biof.ufrj.br (R.M.-B.); 3 Laboratório de Resposta Celular à Infecções Virais, Instituto de Microbiologia Paulo de Góes, Universidade Federal do Rio de Janeiro, Rio de Janeiro 21941-902, RJ, Brazil; danielgavinoleopoldino@gmail.com (D.G.-L.); irispaulaga@gmail.com (I.P.G.-A.); iranaia@micro.ufrj.br (I.A.-M.); 4 Laboratório de Investigação Pulmonar, Instituto de Biofísica Carlos Chagas Filho, Universidade Federal do Rio de Janeiro, Rio de Janeiro 21941-902, RJ, Brazil; ffcruz@biof.ufrj.br; 5 Instituto de Pesquisas de Produtos Naturais, Universidade Federal do Rio de Janeiro, Rio de Janeiro 21941-902, RJ, Brazil; sy2802@gmail.com (S.C.M.); ggleitao@ippn.ufrj.br (G.G.L.); 6 Departamento de Produtos Naturais e Alimentos, Faculdade de Farmácia, Universidade Federal do Rio de Janeiro, Rio de Janeiro 21941-902, RJ, Brazil; sgleitao@pharma.ufrj.br; 7 Faculdade de Química, Escola de Engenharia, Universidade Presbiteriana Mackenzie, São Paulo 01302-907, SP, Brazil; simas.rc@gmail.com

**Keywords:** chikungunya virus, chikungunya fever, triterpenoids, saponins, jujubogenin triglycoside, antivirals

## Abstract

Chikungunya fever, a debilitating disease caused by Chikungunya virus (CHIKV), is characterized by a high fever of sudden onset and an intense arthralgia that impairs individual regular activities. Although most symptoms are self-limited, long-term persistent arthralgia is observed in 30–40% of infected individuals. Currently, there is no vaccine or specific treatment against CHIKV infection, so there is an urgent need for the discovery of new therapeutic options for CHIKF chronic cases. This present study aims to test the antiviral, cytoprotective, and anti-inflammatory activities of an ethanol extract (FF72) from *Ampelozizyphus amazonicus* Ducke wood, chemically characterized using mass spectrometry, which indicated the major presence of dammarane-type triterpenoid saponins. The major saponin in the extract, with a deprotonated molecule ion *m*/*z* 897 [M-H]^−^, was tentatively assigned as a jujubogenin triglycoside, a dammarane-type triterpenoid saponin. Treatment with FF72 resulted in a significant reduction in both virus replication and the production of infective virions in BHK-21-infected cells. The viability of infected cells was assessed using an MTT, and the result indicated that FF72 treatment was able to revert the toxicity mediated by CHIKV infection. In addition, FF72 had a direct effect on CHIKV, since the infectivity was completely abolished in the presence of the extract. FF72 treatment also reduced the expression of the major pro-inflammatory mediators overexpressed during CHIKV infection, such as IL-1β, IL-6, IL-8, and MCP-1. Overall, the present study elucidates the potential of FF72 to become a promising candidate of herbal medicine for alphaviruses infections.

## 1. Introduction

Chikungunya virus (CHIKV) belongs to the *alphavirus* genus of the *Togaviridae* family and consists of one of the most relevant arboviruses of public health concern. Transmitted to humans by the bite of infected *Aedes* spp. mosquitos, CHIKV is widespread across the globe due to the presence of its vectors in both tropical and temperate zones [1]. It is estimated that more than 10 million cases of CHIKV infection occurred in the last decade [2], and this number is supposed to increase in the next years. Infection by CHIKV results in a very debilitating disease, named Chikungunya fever (CHIKF), characterized by a high fever of sudden onset and an intense arthralgia that impairs individual regular activities such as cooking, housecleaning, and others, resulting in a significant social and economic impact [3,4,5]. Although most symptoms are self-limited, long-term persistent arthralgia is observed in 30–40% of infected individuals, and the mechanisms involved in this process are still not understood [6,7]. Mortality rate by CHIKV is relatively low, but recent outbreaks have reported a growing number of fatal cases due to atypical and severe CHIKF manifestations [5], highlighting the importance of continuing to study the mechanisms of CHIKV pathogenesis.

There is still no licensed vaccine or specific treatment against CHIKV infection, and the clinical management of CHIKF relies on pain relief, making the discovery and development of new potential therapeutic options for chronic CHIKF cases urgent. In this context, searching for new pharmacologically active compounds in biodiversity is an interesting and highly successful approach. Natural products are the richest source of structurally and biologically active compounds. Their use was the base of the pharmaceutical industry in the last century and, up to now, continues to be the most optimistic source of molecules to fight against infectious diseases [8,9,10]. Flavonoids and terpenes, two of the most studied classes of natural products, have already been reported to strongly block CHIKV replication [11,12,13,14,15,16,17,18], but several other promising classes are still poorly explored.

Brazil possesses the largest biodiversity in the world, being the habitat of more than 15% of all living species [19]. In addition to the vast source of natural products, Brazil has a cultural tradition of using medicinal plants to treat the most diverse acute and chronic illnesses. An example is *Ampelozizyphus amazonicus* Ducke, a climbing woody liana found in the Amazon rainforest and popularly known as “saracura-mirá”, which is widely used by traditional *quilombola* communities in North Brazil to treat infectious and inflammatory diseases [20]. Serving as an aqueous infusion beverage, known as “cervejinha-de-índio”, it is used to treat and prevent malaria, despite being routinely consumed as a stimulant and tonic [20]. *A. amazonicus* extracts are rich in saponins and free triterpenes [21], the major active compounds responsible for their anti-inflammatory activity [22].

The present study is the first to decipher the antiviral and cytoprotective activity of *A. amazonicus* ethanol extracts, rich in dammarane-type triterpenoid saponins, against CHIKV infection. We found that the saponin-rich extract is able to reduce both the replication of the virus and the production of infective virions in addition to significantly reducing the virus-induced toxicity and the expression of pro-inflammatory mediators. Ethanol extract also had a direct effect on CHIKV particle integrity and showed antiviral activity against Mayaro virus (MAYV), a CHIKV closely related virus, depicturing its usefulness as an anti-alphavirus herbal compound.

## 2. Materials and Methods

### 2.1. Plant Extract

Ethanol extract (FF72) from *A. amazonicus* wood was obtained as previously described [23]. Work with this plant species was authorized by the Brazilian Directing Council of Genetic Heritage (Conselho de Gestão do Patrimônio Genético, CGEN) to access the traditional knowledge associated with bioprospecting purposes, by Resolution CGEN no. 213 (6.12.2007), renewed in Resolution CGEN no. 87/2012, and by the authorization code AEAC3E9 in 2018.

### 2.2. Chemical Characterization of A. amazonicus Ethanol Extract

The LC-APCI-MS analyses were performed using a UHPLC DionexTM UltiMateTM 3000 system with an ultraviolet detector, coupled to LCQ Fleet MS with APCI ionization (ThermoFisher Scientific, Waltham, MA, USA). A Purospher C18 column (2.1 × 150 mm, 2 µ, Merck, Darmstadt, Germany) was used at a flow rate of 0.12 mL/min. High-resolution mass spectrometry (HRMS) analyses were performed using an FT-Orbitrap Q-Exactive (Thermo Fisher Scientific, Waltham, MA, USA) through direct infusion of the diluted samples in MeOH:H_2_O (9:1) containing 0.1% NH_4_OH as a modifier for the negative ionization mode. The MS, equipped with electrospray ionization (ESI) and an Orbitrap analyzer (with 100,000 of resolution), was operated in negative ion mode, as described by [23]. To determine the major compounds of the wood`s ethanol extract, the peak area was calculated using the MZmine 2 v.2.53 software.

### 2.3. Cell Culture

Baby hamster kidney fibroblast (BHK-21-ATCC) cells were cultured in α-MEM (Gibco—Thermo Fisher Scientific, Waltham, MA, USA) with 10% fetal bovine serum (FBS—Sigma-Aldrich, San Luis, MO, USA). Kidney epithelial cells extracted from African green monkey (Vero cells) were cultured in D-MEM High (Gibco) supplemented with 5% non-essential amino acids (Gibco), 5% L-glutamine 200 mM (Gibco), and 10% FBS. Both cells were cultured in a humidified chamber at 37 °C with 5% carbon dioxide (CO_2_). Chikungunya Virus (RJ5 strain) [24] was propagated according to a previously described method [25]. A supernatant obtained from a non-infected cell culture was used as the mock control.

### 2.4. BHK-21 Infection and FF72 Treatment

BHK-21 cells were cultured in α-MEM with 10% FBS until reaching 80% confluence. The medium was discarded, and the cells were washed twice with PBS 1× pH 7.4 prior to infection with CHIKV, MAYV, or Mock at a multiplicity of infection (MOI) of 0.2 in α-MEM without FBS for 2 h. After this period, the medium was discarded, and a fresh medium containing either 25, 50, or 100 μg/mL of FF72 or vehicle was added to the cell culture prior to incubation for 24 or 72 h at 37 °C with 5% CO_2_. For the direct effect of FF72 on CHIKV, the 10^6^ plaque forming unit (PFU) of CHIKV was incubated with either 25, 50, or 100 μg/mL of FF72 or vehicle for 2 h at 37 C. Infectious particles were then quantified using a plaque assay.

### 2.5. Cell Viability using the MTT Assay

BHK-21 cells were cultured in α-MEM supplemented with 10% FBS until reaching 80% confluence in a 96-well plate. The medium was discarded, and a fresh α-MEM with 10% FBS containing 25, 50, 125, or 250 μg/mL of FF72 was added to the cells, followed by incubation for 72h at 37 °C under 5% CO_2_. After this period, 100 µL of MTT [3-(4,5-dimethylthiazolyl-2)-2,5-diphenyltetrazolium bromide] (Sigma Aldrich) was added to each sample and incubated at 37 °C with 5% CO_2_ for 4h followed by the addition of 50 µL of dimethyl sulfoxide (DMSO [Sigma Aldrich]), and incubation occurred for 30 min at 37 °C with 5% CO_2_ to dissolve formazan crystals. The absorbance was read at 570 nm in a plate reader. The absorbance values were converted into the percentage of metabolically viable cells relative to the respective control group.

### 2.6. Virus Quantification Using Plaque Assay

The supernatant of CHIKV or MAYV-infected BHK-21 cells (treated or not) or CHIKV particles previously incubated with vehicle 25, 50, or 100 μg/mL of FF72 for 2 h at 37 °C were serially diluted (10-fold) in high-glucose DMEM without FBS. Each dilution was used to infect confluent Vero cells seeded in 24-well plates. After 1 h of adsorption at 37 °C in a humidified 5% CO_2_ chamber, the medium was removed, and 1 mL/well of overlay medium (3% carboximethylcellulose [CMC-Sigma-Aldrich] and DMEM High 2X with 2% FBS) was added, incubated at 37 °C with 5% CO_2_ for 48h, and then fixed using a 10% formaldehyde solution for 20 min. The volume was discarded and the cells were stained with crystal violet solution (1% Crystal Violet in 20% ethanol) for 30 min at room temperature. The plaques were counted, and virus load was expressed as pfu/mL.

### 2.7. Virus Quantification and Cytokine Expression by RT-qPCR

Total RNA from CHIKV-infected BHK-21 cells treated with vehicle or FF72 was extracted using the PureLink RNA mini kit (Thermo Fisher Scientific). The RNA was converted into cDNA with the High-Capacity cDNA Reverse Transcription Kit (Thermo Fisher Scientific). The quantification of the CHIKV genome was performed with 100 ng of cDNA and 300 ng of the following primer (5′ 6-FAM/CGCTGTGATACAGTGGTTTCGTGTG/BHQ-1 3′) on the Applied System Biosystems 7500 RT—PCR device using the TaqMan Mix kit (Thermo Fisher Scientific) according to the manufacturer’s instructions. A standard curve established from a serial dilution of the viral stock was used to determine virus load, as previously described by [26]. Interleukin (IL)-6, IL-8, monocyte chemoattractant protein (MCP)-1, and IL-1β mRNA expressions were measured with 100 ng of cDNA and 300 ng of each pair of primers (Table 1) on the Applied System Biosystems 7500 RT—PCR device using the SYBR Green PCR Master Mix kit (Thermo Fisher Scientific) according to the manufacturer’s instructions. For each sample measured in duplicate, gene expression was normalized to that of the housekeeping gene (acidic ribosomal phosphoprotein P0, 36B4) and expressed as the fold change relative to the control group using the 2^−ΔΔCt^ method.

### 2.8. Statistical Analysis

Data were tested for normality using the Kolmogorov–Smirnov test with Lilliefors’ correction, and the Levene median test was used to evaluate the homogeneity of the variances. The data satisfied the parametric assumptions and were expressed as the mean and standard deviation (SD). Comparisons were made using an unpaired *t* test. All calculations were performed using GraphPad Prism version 9.00 for Windows (GraphPad Software, San Diego, CA, USA), and p values below 0.05 were considered significant.

## 3. Results

### 3.1. Chemical Characterization of the Major Compounds of the Ethanol Extract from A. amazonicus Ducke Wood

**Figure 1:**
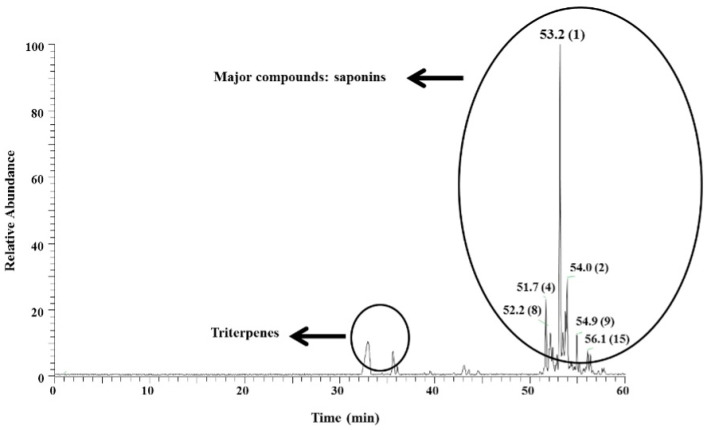
UHPLC-MS/MS chromatogram of the wood ethanol extract FF72.

The ethanol extract of *A. amazonicus* (FF72) was previously investigated by combined strategies of tandem mass spectrometry (UHPLC-MS/MS) for the analysis of fragmentation patterns, as well as direct sample injection and electrospray ionization combined with high-resolution mass spectrometry (DI-ESI-HRMS) measurements, allowing for the identification of 92 dammarane-type triterpenoid saponins in FF72 [23]. In this work, the saponin profile of the extract was investigated by UHPLC-MS/MS using the APCI ionization in negative ion mode. The chromatogram (Figure 1) shows a complex saponin profile which is also composed of free triterpenes. Based on the peak area calculation from the UHPLC-MS/MS data, it was possible to identify 15 main saponins, all of them with a dammarane-type triterpenoid aglycone. The most abundant in FF72 extract, with a deprotonated molecule ion of *m*/*z* 897 [M-H]^−^ (saponin 1, Rt 53.2 min), was tentatively assigned as a jujubogenin triglycoside, a dammarane-type triterpenoid saponin (Table 2).

### 3.2. FF72 Does Not Induce Cell Toxicity

**Figure 2:**
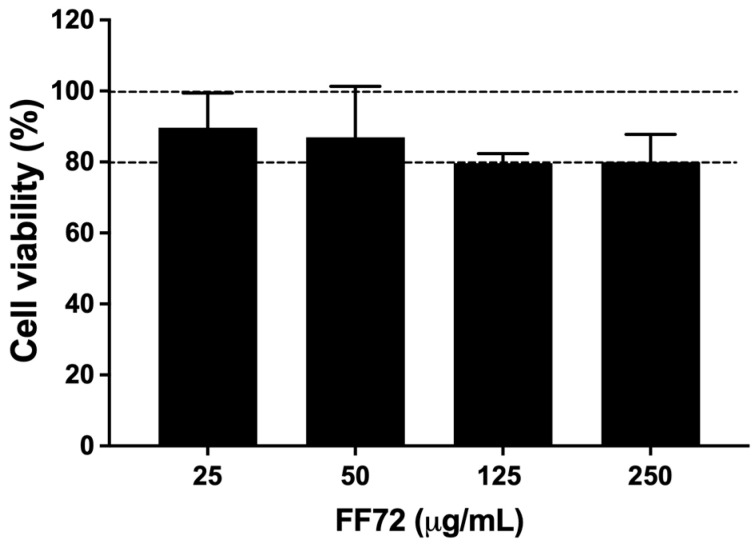
Cell viability assessed using MTT assay. BHK-21 cells were treated with FF72 extract (25 to 250 μg/mL) for 24 h prior to MTT analysis. Percentage of cell viability was calculated based on conditions in which cells were treated with vehicle. Error bars correspond to standard deviation of 4 independent experiments.

Prior to evaluating the pharmacological role of FF72, safety was first assessed by testing whether it induced cell cytotoxicity. BHK-21 cells were used as the cell model for both the cytotoxicity and pharmacological activity measurements since these cells are a well-established model in the literature for CHIKV replication studies [27], in addition to reproducing most of the mechanisms observed in synovial fibroblasts, which are one of the most important cell targets of CHIKV infection in humans [27]. Cytotoxicity was measured after the incubation of different concentrations of FF72 into BHK-21 cells for 72 h. The results observed in Figure 2 indicate that cell viability remained above 80% compared to vehicle-treated cells (100% of cell viability) in all the conditions tested. FF72 has a 50% cytotoxic concentration (CC_50_) above 250 μg/mL, which suggests that it does not induce significant cell toxicity up to this concentration.

### 3.3. Antiviral Effect of FF72 on CHIKV Infection

**Figure 3:**
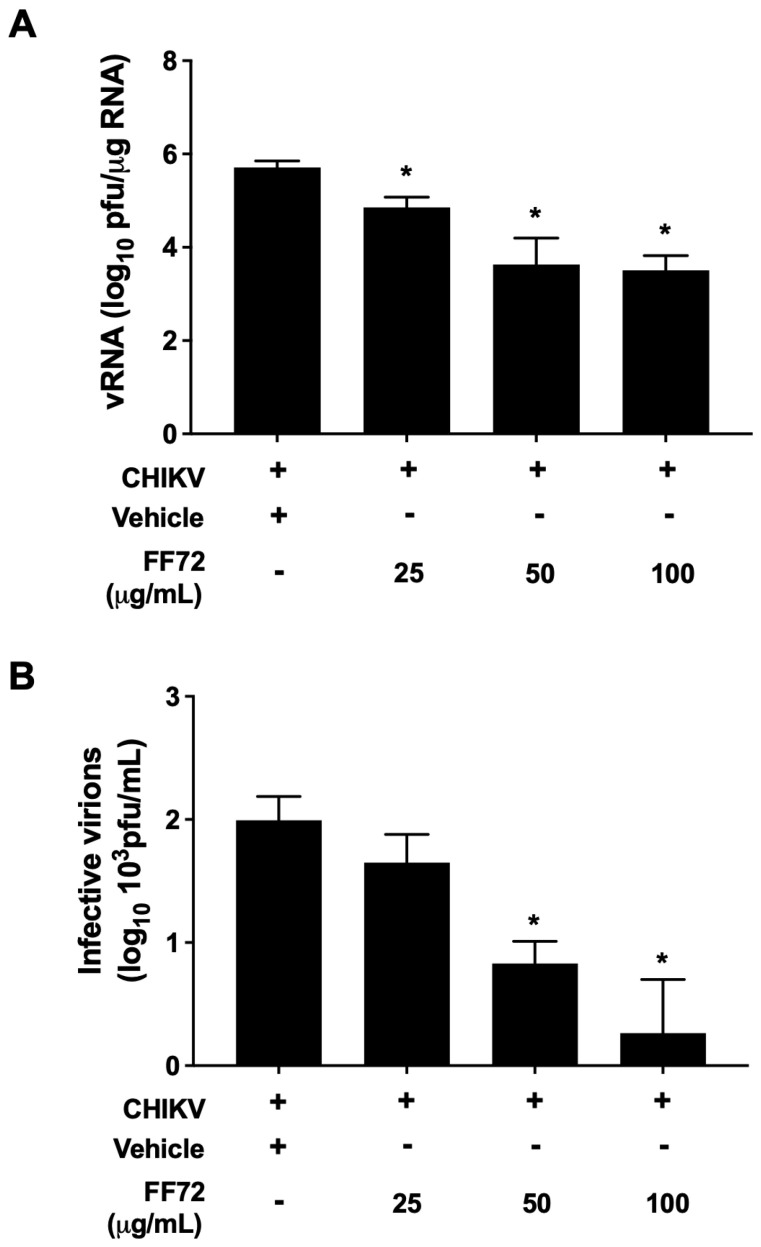
Antiviral effect of FF72 extract. BHK-21 cells were infected at an MOI of 0.2 in the absence or presence of different concentrations of FF72 for 24 h. After this period, total RNA was extracted and vRNA was measured using RT-qPCR. The amount of vRNA was calculated based on a standard curve of CHIKV RNA (**A**). The supernatant of the cells was collected and subjected to a plaque assay for measuring the amount of infective viral particles released (**B**). Statistical significance was calculated using a nonparametric Student *t* test, in which * represents *p* values lower than 0.05 compared to vehicle-treated condition.

A reduction in both virus replication and the production of infective virions is the main goal of any antiviral candidates. In this context, to evaluate whether FF72 extract plays an antiviral role, we first assessed the amount of CHIKV genomic RNA (vRNA) replication in BHK-21 cells infected with CHIKV RJ5 strain using a standardized RT-qPCR [26]. Treatment with either 25, 50, or 100 µg/mL of FF72 was able to significantly reduce CHIKV vRNA replication (Figure 3A) at a 50% efficacy concentration (EC_50_) of 19.95 µg/mL. It is worth noting that FF72 treatment in higher concentrations reduced vRNA in up to 2 logs, which reflects an outstanding reduction in the intracellular viral load. To discover whether FF72 extract is also capable of decreasing the production of new infective particles, the supernatant of infected BHK-21 cells was subjected to a plaque assay. A significant reduction in the production of infective virions was achieved after treatment with 50 and 100 µg/mL of FF72 (Figure 3B). Treatment with 50 µg/mL reduced both virus replication and the production of infective virions by 2 and 1 log, respectively. The same effect was not observed after treatment with 25 ug/mL, which resulted in a reduction by 1 log of viral RNA, and there was no effect on the formation of infective particles (Figure 3A). Interestingly, treatment with the highest dose led to a reduction in the infective virions titer of at least 2 logs, which is similar to the observed reduction in vRNA, indicating that at higher concentrations, the extract impacts both the virus RNA replication and the production of new infective particles.

**Figure 4:**
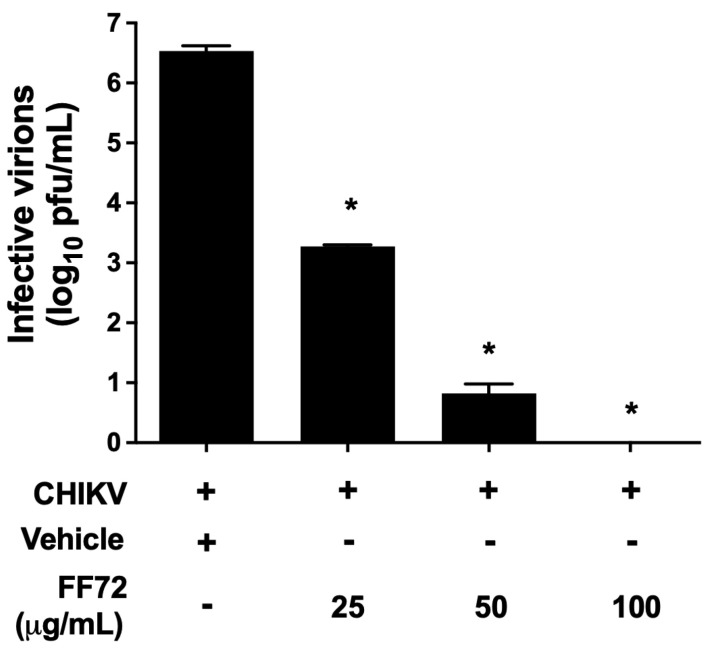
Evaluation of a direct effect of FF72 on CHIKV. Around 2.0 × 10^6^ pfu/mL of CHIKV was incubated with vehicle or different concentrations of FF72 for 2 h at 37 °C prior to a plaque assay. The reduction in the amount of infective virions compared to the vehicle-treated control reflects the ability of FF72 extract to directly impair CHIKV to infect new cells. Statistical significance was calculated using a nonparametric Student *t* test, in which * represents *p* values lower than 0.05 compared to vehicle-treated condition.

Saponins, the major components of FF72 extract, are largely recognized by their amphipathic characteristic and their ability to modulate membrane structure and function. Therefore, to understand whether the extract exerts a direct effect on CHIKV envelope, 10^6^ pfu of CHIKV was incubated with vehicle or FF72 at different concentrations for 2 h at 37 °C, followed by titration using a plaque assay. Incubation with FF72 was able to significantly decrease CHIKV infectivity at an EC_50_ of 0.0105 µg/mL (Figure 4). At 25 µg/mL, the treatment reduced CHIKV infectious particles by approximately 3 logs compared to the negative control. This effect was more pronounced at 50 µg/mL of FF72, in which a reduction in infective virions of at least 5 logs was observed. Surprisingly, the incubation with 100 µg/mL of FF72 completely abolished the capacity of CHIKV to infect cells. These results strongly indicate that FF72 presents a direct effect on circulating viral particles, impairing their ability to infect naive cells.

### 3.4. Cytoprotective Role of FF72 Extract

**Figure 5:**
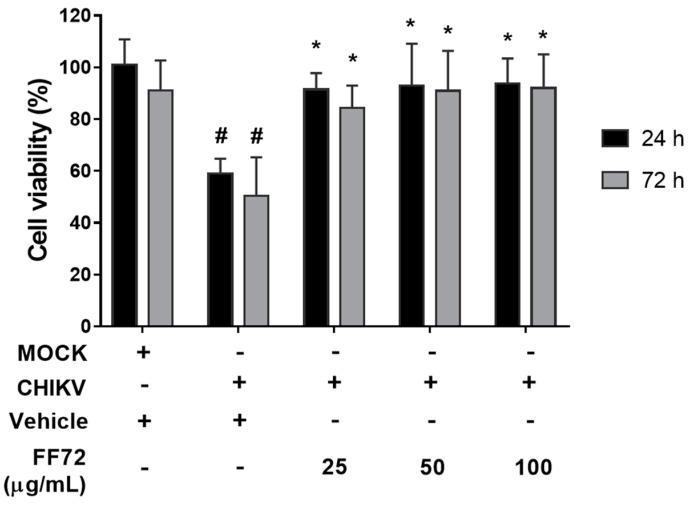
Assessment of virus-induced cell cytotoxicity. BHK-21 cells were infected at an MOI of 0.2 in the absence or presence of different concentrations of FF72 for 24 h or 72h. At the end of this period, cell viability was assessed using MTT assay. Percentage of cell viability was calculated based on conditions in which cells were not treated. Statistical significance was calculated using a nonparametric Student *t* test, in which # represents *p* values lower than 0.01 compared to vehicle-treated condition and * represents *p* values lower than 0.05 compared to CHIKV condition treated with vehicle.

It is largely known that virus infection modulates cell metabolism to its own benefit, which generally results in an increase in host cell cytotoxicity. CHIKV-induced toxicity in BHK-21 cells was monitored using an MTT assay and, as observed in Figure 5, CHIKV reduces cell viability by approximately 40 and 50% after 24 and 72 h infection, respectively. To assess whether FF72 extract exerts a cytoprotective role to BHK-21 cells, the cells were infected with CHIKV followed by treatment with FF72 at different concentrations for 24 and 72 h prior to the analysis using MTT. FF72 treatment prevented virus-induced cell toxicity in all the conditions tested and in both time points (Figure 5). The viability of the infected cells in the presence of FF72 extract was similar to that observed in the non-infected condition, indicating that this extract also has the potential do avoid CHIKV-induced cell damage.

### 3.5. Anti-Inflammatory Role of FF72 Extract

**Figure 6:**
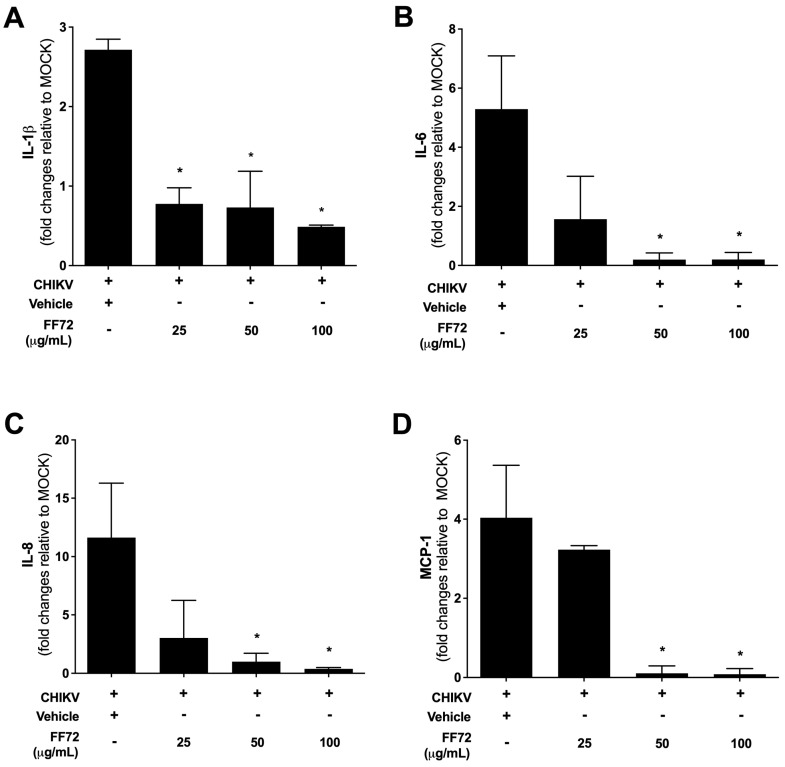
Assessment of cytokine expression using RT-qPCR. BHK-21 cells were infected at an MOI of 0.2 in the absence or presence of different concentrations of FF72 for 24 h. At the end of this period, total RNA was extracted, and expression levels of IL-1β (**A**), IL-6 (**B**), IL-8 (**C**), and MCP-1 (**D**) were determined using qRT-PCR. Statistical significance was calculated using a nonparametric Student *t* test, in which * represents *p* values lower than 0.05 compared to vehicle-treated condition.

The overexpression of inflammatory cytokines is a key event during CHIKV infection since it contributes to the establishment and maintenance of severe arthralgia observed in most infected patients. A strong increase in TNFα, IL-1β, IL-6, IL-8, and MCP-1 levels is largely reported in the literature and is the main factor that drives cartilage damage (as reviewed by [27]). Therefore, to evaluate whether BHK-21 can be used as a model for studying CHIKV-induced inflammation, the expression levels of the most relevant pro-inflammatory cytokines were assessed using qRT-PCR. CHIKV infection induces the overexpression of IL-1β, IL-6, IL-8, and MCP-1 in BHK-21 cells, confirming that these cells consist of a good model for studying the contribution of non-immune cells to the CHIKV-induced pro-inflammatory mediator (Figure 6).

Based on this, the expression levels of those cytokines were evaluated in CHIKV-infected BHK-21 cells after treatment with different concentrations of FF72. The treatment significantly reduced the expression of IL-1β in all the conditions tested (Figure 6A), but a significant reduction in the IL-6 (Figure 6B), IL-8 (Figure 6C), and MCP-1 (Figure 6D) levels was only achieved after treatment with the highest concentrations of FF72 (50 and 100 µg/mL). These results clearly indicate that FF72 treatment prevents the pro-inflammatory overexpression induced by CHIKV infection [22].

### 3.6. Antiviral Effect of FF72 against MAYV

**Figure 7:**
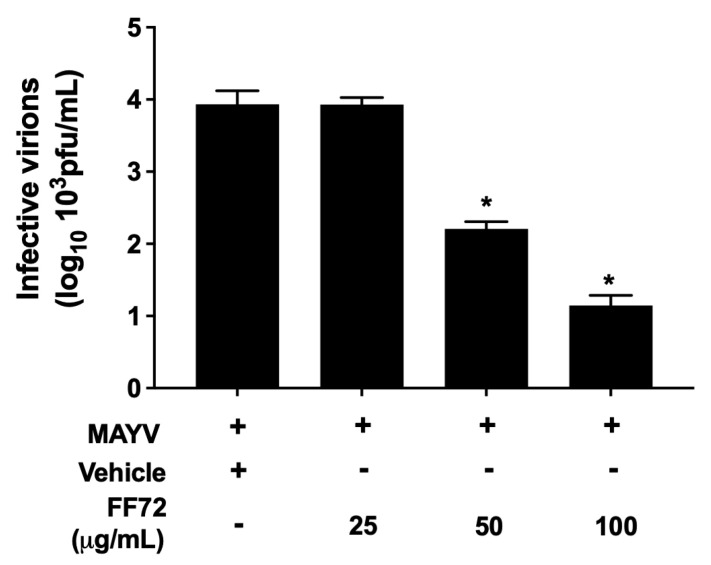
Antiviral effect of FF72 extract on MAYV. BHK-21 cells were infected with MAYV at an MOI of 0.2 in the absence or presence of different concentrations of FF72 for 24 h. The supernatant of the cells was collected and subjected to a plaque assay for measuring the amount of infective virions. Statistical significance was calculated using a nonparametric Student *t* test, in which * represents *p* values lower than 0.05 compared to vehicle-treated condition.

Considering the expressive antiviral activity of FF72 extract, it was further assessed whether it is also observed to be closely related to other arboviruses. To answer this question, BHK-21 cells were infected with MAYV, an arthritogenic alphavirus of the same CHIKV family, at the same conditions described previously. The result observed in Figure 7 indicates that FF72 exhibits an antiviral activity to MAYV, in a similar fashion to that observed for CHIKV, with an EC_50_ of 28.03 μg/mL. FF72 treatment with 50 µg/mL reduced in about 2 logs the titer of infective virions, and 100 µg/mL reduced it by over 3 logs. Treatment with 25 µg/mL did not exert any effect on virion production. Overall, these data clearly indicate the anti-alphavirus activity of FF72 extract.

## 4. Discussion

Currently, several CHIKV vaccine candidates as well as antiviral compounds are in clinical trials, but none have been approved to market so far. CHIKF is clinically managed with analgesics and non-steroidal anti-inflammatory drugs (NSAIDs), but chronic patients, who are usually nonresponsive to these options, are treated with corticoids and, eventually, with disease-modifying anti-rheumatic drugs (DMARDs), which are potent immunosuppressive drugs with a long list of side effects and controversial responsiveness [28]. Therefore, the investigation of new molecular entities with prominent antiviral activity is of extreme importance in the context of alphavirus infection. In the present study, we explore the antiviral, cytoprotective, and anti-inflammatory role played by dammarane-type triterpenoid saponins extracted from *A. amazonicus* Ducke, a climbing woody liana found in the Amazon rainforest, against CHIKV.

Saponins, the major components of FF72 extract, are a class of secondary metabolites with a high molecular weight (from 600 to 2000 Da), widely distributed in nature, and possessing both great diversity and structural complexity. Saponins are glycosides composed by an aglycone portion (hydrophobic character), also called sapogenin, and a glycidic portion (hydrophilic character), making them amphipathic molecules. Saponins have a unique ability to produce abundant and persistent foam when shaken in aqueous solution [29]. When in contact with living cells, this class of molecules exhibits an excellent surfactant activity, in addition to their ability to interact with different classes of biological molecules. In this regard, saponins exhibit cytotoxic/antitumor, hemolytic, hepatoprotective, immunostimulant, antimicrobial, and anti-inflammatory properties [30]. More recently, saponins have been described to inhibit SARS-CoV-2 infection [31], and the anti-SARS-CoV-2 activity of aqueous and ethanol bark extracts from *A. amazonicus* has been recently described [32]. However, the literature regarding their activity against other viruses of public health concern is very scarce.

Due to saponins’ detergent-like activity, most of their biological activities are explained by their ability to bind phospholipids and cholesterol in the cell membrane, altering membrane stability and inducing cell lysis [30]. Although some saponin-rich extracts are extremely toxic to cells and are generally explored as larvicidal agents [33,34,35], our data show that FF72 saponin-rich extract is quite safe to cells, with a selective index (SI) above 12.53. Interestingly, our data clearly indicate that it rather exhibits a cytoprotective activity, preventing virus-induced cell death. On the other hand, FF72 hampered CHIKV particle integrity in such a way that it completely abolished virus adsorption and infection, suggesting a dual and antagonist role being played in the cells and virus in terms of membrane integrity.

In addition to escape from host immune responses, successful virus infection should accomplish three major steps: (i) adsorption into target cell and internalization, (ii) genome replication, and (iii) the production of new infective virions [27]. In principle, any safe compound able to direct or indirectly block at least one of the aforementioned steps has the potential to become an antiviral candidate. To our surprise, FF72 impaired virus infection (direct effect on virus particle) and significantly reduced vRNA and virion formation, confirming its outstanding antiviral activity. Natural products, as the richest source of structurally diverse and pharmacologically active compounds, have already been investigated for their activity against CHIKV, and several compounds and extracts have been reported as promising antivirals [11,12,13,14,15,16,17,18], proving the relevant contribution of natural products in the fight against life-threating viruses. Saponins’ antiviral activity was also assessed before, where they were described to interact with virus capsid protein and change the integrity of the membrane of infected cells [36,37,38]. A previous study from [39] reported the antiviral potential of saponin fractions from *Quillaja* spp. against CHIKV, which corroborates with the data we are presenting here. We also exploited the ability of FF72 to act against MAYV, an arthritogenic virus closely related to CHIKV. Our data show that it is also very effective at reducing the formation of MAYV virions, making our extract a promising anti-arthritogenic herbal medicine.

The overexpression of inflammatory cytokines is a key event during CHIKV infection since it contributes to the establishment and maintenance of severe arthralgia observed in most infected patients. Strong increases in TNFα, IL-1β, IL-6, IL-8, and MCP-1 levels are largely reported in the literature, and this is the main factor that drives cartilage damage (as reviewed by [27]). Since the anti-inflammatory activity of saponin-rich extracts from *A. amazonicus* has already been reported [21,22], we also investigated whether FF72 was able to reduce the expression of inflammatory mediators produced by CHIKV. Indeed, our data indicate that FF72 also exerts an anti-inflammatory activity against CHIKV, either indirectly as a consequence of the reduction in virus activity, or directly by a still undescribed mechanism.

Overall, our study depicts the multifunctional activity of the *A. amazonicus* ethanol extract rich in dammarane-type triterpenoid saponins against CHIKV infection. We believe that our data will pave the way for further studies aiming to develop novel herbal drugs from Amazonian biodiversity against an important virus of public health concern.

## Figures and Tables

**Table 1:** Primer sequences.

Gene	Forward	Reverse
CHIKV	5′ AAAGGGCAAACTCAGCTTCAC 3′	5′ GCCTGGGCTCATCGTTATTC 3′
IL-6	5′ CTGCAAGAGACTTCCATCCAG 3′	5′AGTGGTATAGACAGGTCTGTTGG 3′
IL-8	5′ ATGACTTCCAAGCTGGCCGTGGCT 3′	5′ TCTCAGCCCTCTTCAAAAACTTCTC 3′
MCP-1	5′ GCATCCACGTGTTGGCTCA 3′	5′ CTCCAGCCTACTCATTGGGATCA 3′
IL-1β	5′ TTCAGGCAGGCAGTATCACTC 3′	5′ CCACGGGAAAGACACAGGTAG 3′
36b4	5′ CGACCTGGAAGTCCAACTAC 3′	5′ ATCTGCTGCATCTGCTTG 3′

**Table 2:** UHPLC-MS/MS data of the 15 main saponins found in the ethanol extract of *A. amazonicus* wood (FF72).

ID (Saponins)	RT (min)	M-H (*m*/*z*)	Molecular Formula	Sugar Residue
1	53.2	897.5	C_46_H_74_O_17_	1 Hex, 2 Pen
2	54.0	787.5	C_43_H_64_O_13_	1 Hex, 1 dHex
3	53.8	773.5	C_42_H_62_O_13_	1 Hex, 1 Pen
4	51.7	1059.3	C_52_H_84_O_22_	2 Hex, 2 Pen
5	53.1	1073.4	C_53_H_86_O_22_	2 Hex, 1 dHex, 1 Pen
6	53.6	1115.3	C_55_H_88_O_23_	2 Hex, 1 Pen, 1 dHex
7	52.2	915.3	C_46_H_76_O_18_	1 Hex, 2 Pen
8	53.7	911.4	C_47_H_76_O_17_	1 Hex, 1 dHex, 1 Pen
9	54.9	969.3	C_49_H_78_O_19_	2 Hex, 1 Pen
10	52.8	927.5	C_47_H_76_O_18_	2 Hex, 1 Pen
11	53.8	929.3	C_47_H_78_O_18_	1 Hex, 1 dHex, 1 Pen
12	53.9	943.2	C_48_H_80_O_18_	1 Hex, 1 dHex, 1 Pen
13	52.4	1103.3	C_54_H_88_O_23_	3 Hex, 1 dHex
14	52.9	949.4	C_49_H_74_O_18_	2 Hex, 1 dHex
15	56.1	973.5	C_49_H_82_O_19_	2 Hex, 1 Pen

## Data Availability

The data presented in this study are available on request from the corresponding author.
